# The Pyrroloquinoline-Quinone-Dependent Pyranose Dehydrogenase from Coprinopsis cinerea Drives Lytic Polysaccharide Monooxygenase Action

**DOI:** 10.1128/AEM.00156-18

**Published:** 2018-05-17

**Authors:** Anikó Várnai, Kiwamu Umezawa, Makoto Yoshida, Vincent G. H. Eijsink

**Affiliations:** a Faculty of Chemistry, Biotechnology and Food Science, Norwegian University of Life Sciences (NMBU), Ås, Norway; b Department of Environmental and Natural Resource Science, Tokyo University of Agriculture and Technology, Tokyo, Japan; Goethe University Frankfurt am Main

**Keywords:** PQQ-dependent PDH, AA12, carbohydrate-binding module, lytic polysaccharide monooxygenase, LPMO, AA9, electron transfer

## Abstract

Fungi secrete a set of glycoside hydrolases and oxidoreductases, including lytic polysaccharide monooxygenases (LPMOs), for the degradation of plant polysaccharides. LPMOs catalyze the oxidative cleavage of glycosidic bonds after activation by an external electron donor. So far, only flavin-dependent oxidoreductases (from the auxiliary activity [AA] family AA3) have been shown to activate LPMOs. Here, we present LPMO activation by a pyrroloquinoline-quinone (PQQ)-dependent pyranose dehydrogenase (PDH) from Coprinopsis cinerea, *Cc*PDH, the founding member of the recently discovered auxiliary activity family AA12. *Cc*PDH contains a C-terminal family 1 carbohydrate binding module (CBM1), an N-terminal family AA8 cytochrome domain, and a central AA12 dehydrogenase domain. We have studied the ability of full-length *Cc*PDH and its truncated variants to drive catalysis by two Neurospora crassa LPMOs. The results show that *Cc*PDH indeed can activate the C-1-oxidizing N. crassa LPMO 9F (*Nc*LPMO9F) and the C-4-oxidizing Neurospora crassa LPMO 9C (*Nc*LPMO9C), that this activation depends on the cytochrome domain, and that the dehydrogenase and the LPMO reactions are strongly coupled. The two tested *Cc*PDH-LPMO systems showed quite different efficiencies, and this difference disappeared upon the addition of free PQQ acting as a diphenol/quinone redox mediator, showing that LPMOs differ when it comes to their direct interactions with the cytochrome domain. Surprisingly, removal of the CBM domain from *Cc*PDH had a considerable negative impact on the efficiency of the *Cc*PDH-LPMO systems, suggesting that electron transfer in the vicinity of the substrate is beneficial. *Cc*PDH does not oxidize cello-oligosaccharides, which makes this enzyme a useful tool for studying cellulose-oxidizing LPMOs.

**IMPORTANCE** Lytic polysaccharide monooxygenases (LPMOs) are currently receiving increasing attention because of their importance in degrading recalcitrant polysaccharides and their potential roles in biological processes, such as bacterial virulence. LPMO action requires an external electron donor, and fungi growing on biomass secrete various so-called glucose-methanol-choline (GMC) oxidoreductases, including cellobiose dehydrogenase, which can donate electrons to LPMOs. This paper describes how an enzyme not belonging to the GMC oxidoreductase family, *Cc*PDH, can activate LPMOs, and it provides new insights into the activation process by (i) describing the roles of individual *Cc*PDH domains (a dehydrogenase, a cytochrome, and a carbohydrate-binding domain), (ii) showing that the PDH and LPMO enzyme reactions are strongly coupled, (iii) demonstrating that LPMOs differ in terms of their efficiencies of activation by the same activator, and (iv) providing indications that electron transferring close to the substrate surface is beneficial for the overall efficiency of the *Cc*PDH-LPMO system.

## INTRODUCTION

Plant biomass is a promising raw material for industrial use because of its abundance in nature. However, industrial utilization of biomass via a biochemical conversion route is limited due to its resistance to enzymatic degradation (1). The recalcitrance of plant biomass resides in the plant cell wall, which is an insoluble and partly crystalline copolymeric structure composed of cellulose, hemicellulose, and lignin. The homopolymeric cellulose chains are organized into highly crystalline cellulose bundles, called cellulose microfibrils (2). Due to their compact structure, cellulose microfibrils are resistant to enzymatic depolymerization. Plant cell wall-degrading fungi, which are the main plant biomass decomposers in nature, express a set of glycoside hydrolases and oxidoreductases, including lytic polysaccharide monooxygenases (LPMOs), for the degradation of cellulose and other plant cell wall polysaccharides (3, 4).

LPMOs are copper-dependent enzymes that catalyze the oxidative cleavage of glycosidic bonds in various polysaccharides, such as cellulose (5–7), hemicelluloses (8–12), chitin (13), and starch (14). The known diversity in the substrate specificities of LPMOs and the abundances of LPMO-encoding genes in many microbes indicate that these enzymes are important in degradation of various biomasses (3, 4). LPMOs are currently classified into auxiliary activities (AA) families 9, 10, 11, 13, 14, and 15 in the Carbohydrate-Active enZymes (CAZy) database, which is based on the similarity of amino acid sequences (15). Fungal LPMOs belonging to the family AA9, also known as LPMO9s, contribute to the degradation of cellulose and hemicelluloses. Since these LPMOs accelerate the hydrolysis of cellulose by cellulases, they have recently attracted considerable attention as a key enzyme in efficient cellulose saccharification processes (16–19).

During its catalytic cycle, an LPMO requires an external electron donor for (re)activation (13, 20, 21). The electron donor can be a nonenzymatic reducing compound, such as ascorbic acid, lignin, and other plant biomass-derived phenols (13, 20, 22, 23). Alternatively, LPMOs can be activated by flavin-dependent oxidoreductases, directly or through plant-derived diphenols and quinones acting as redox mediators (17, 20, 24, 25). Flavoenzymes that have been shown to supply LPMOs with electrons are classified into the CAZy family AA3 and include cellobiose dehydrogenase (CDH; AA3_1), glucose dehydrogenase (AA3_2), and aryl-alcohol quinone oxidoreductase (AA3_2) (17, 20, 24–26). CDH, the first enzyme reported to be able to activate an LPMO (7, 17), is an extracellular flavocytochrome consisting of two domains: an N-terminal *b*-type heme-binding AA8 cytochrome domain and a C-terminal flavin adenine dinucleotide (FAD)-dependent AA3_1 dehydrogenase domain (27). Some CDHs have an additional family 1 carbohydrate-binding module (CBM1) at their C terminus (28). The AA3_1 dehydrogenase domain oxidizes cellobiose at the C-1 position to cellobiono-1,5-lactone, or longer cello-oligosaccharides to the corresponding lactones, via the reduction of the FAD cofactor (29). Then, in one scenario, two electrons from the FAD cofactor are sequentially transferred to an acceptor via the heme of the AA8 cytochrome *b* domain, which carries out two subsequent single-electron transfers (24, 30, 31). The external electron acceptor could be, for example, cytochrome *c* or an LPMO (24, 26, 31). In an alternative slower scenario, the FAD is reoxidized by direct electron transfer to molecular oxygen, leading to the production of hydrogen peroxide (20, 24, 32).

Recently, a new AA family, AA12, has been discovered, with the founding member being the pyrroloquinoline quinone (PQQ)-dependent pyranose dehydrogenase (PDH) from the plant saprophytic basidiomycete Coprinopsis cinerea (*Cc*PDH), which is the first PQQ-dependent enzyme in eukaryotes (33). This enzyme has a three-domain structure similar to some CDHs. It is composed of an N-terminal heme *b*-binding AA8 cytochrome domain, a central PQQ-dependent AA12 dehydrogenase domain (instead of the AA3_1 cellobiose-oxidizing flavin domain in CDH), and a C-terminal CBM1. The PQQ cofactor binds with high affinity, and the binding constant, *K_d_*, is 1.1 nM (33). The PQQ-dependent PDH domain shows oxidative activity toward d-glucosone (2-keto–d-glucose), l-fucose, and some rare pyranoses but is inactive toward glucose and cellobiose (33, 34). Of note, d-glucosone is the product of glucose oxidation by pyranose-2-oxidase, an enzyme found in several fungi. The N-terminal cytochrome domain of *Cc*PDH exhibits 39% sequence identity with the cytochrome domain of *Pc*CDH from the wood-rotting basidiomycete Phanerochaete chrysosporium, and spectral features, such as UV-Vis and resonance Raman spectra, are almost identical for *Cc*PDH and *Pc*CDH (34). The redox potentials of the cytochrome domains of the two enzymes are identical (130 mV, pH 7, versus standard hydrogen electrode [SHE]) (34, 35), indicating that, like CDH, *Cc*PDH could also transfer electrons to LPMOs (E^o^ on the order of 250 mV versus SHE [20]).

Although there is no known role for *Cc*PDH in plant cell wall degradation, such a role is suggested by the presence of the CBM1 and by the fact that the gene adjacent to the *Cc*PDH-encoding gene encodes an LPMO (gene identification [ID], CC1G_09526). In the present study, we investigated the potential of *Cc*PDH to drive catalysis by N. crassa LPMO 9C and N. crassa LPMO 9F (*Nc*LPMO9C and *Nc*LPMO9F, respectively), two well-characterized LPMOs from the ascomycete Neurospora crassa, and we compared *Cc*PDH with ascorbic acid as an electron donor. Since, in contrast to CDH, *Cc*PDH is inactive toward glucose and cello-oligosaccharides, reducing end-containing LPMO-generated products released from the cellulosic substrate are not oxidized by *Cc*PDH. Thus, *Cc*PDH is a good candidate to study the activity and product profiles of cellulose-active LPMOs. Furthermore, as shown here, product formation by *Cc*PDH can be analyzed chromatographically, simultaneously with the analysis of LPMO-generated products, generating detailed insights into how the two enzymes work together.

## RESULTS

### Activation of LPMO9s by *Cc*PDH.

In a previous study (36), we showed that the PQQ-dependent 2-keto–d-glucose dehydrogenase (2KGDH) from Pseudomonas aureofaciens, a bacterial homolog of *Cc*PDH, oxidizes the aldehyde group of d-glucosone at the C-1 position, yielding 2-keto-d-gluconic acid, which is a precursor of erythorbic acid (also called isoascorbic acid). In the present study, we used external standards to identify the products generated by *Cc*PDH from d-glucosone and l-fucose. The retention times of the reaction products generated from d-glucosone and l-fucose were 16.6 min and 8.9 min, i.e., identical to the retention times of 2-keto-d-gluconic acid and l-fuconic acid, respectively (see Fig. S1 in the supplemental material). Thus, as expected, *Cc*PDH oxidizes the C-1 position of these substrates. In order to exclude the possibility that LPMOs may be activated by these reaction products of *Cc*PDH, we incubated LPMOs with 2-keto-d-gluconic acid, l-fuconic acid, or products generated by *Cc*PDH from after enzyme inactivation by boiling. None of these compounds were able to activate the LPMOs (Fig. S2 and S3). The control experiments depicted in Fig. S1 and S2 also showed that quantification of PDH action is possible in the case of d-glucosone as the substrate, since the oxidized product, 2-keto-d-gluconic acid, gives a clear sharp peak with a good response factor in the required concentration range (Fig. S1A). In the case of l-fucose, detection of l-fuconic acid was not sensitive enough in the required concentration range. In principle, the sharp l-fucose peak with a high response factor (Fig. S3A) would allow for the quantification of PDH action through measuring l-fucose consumption.

**FIG 1:**
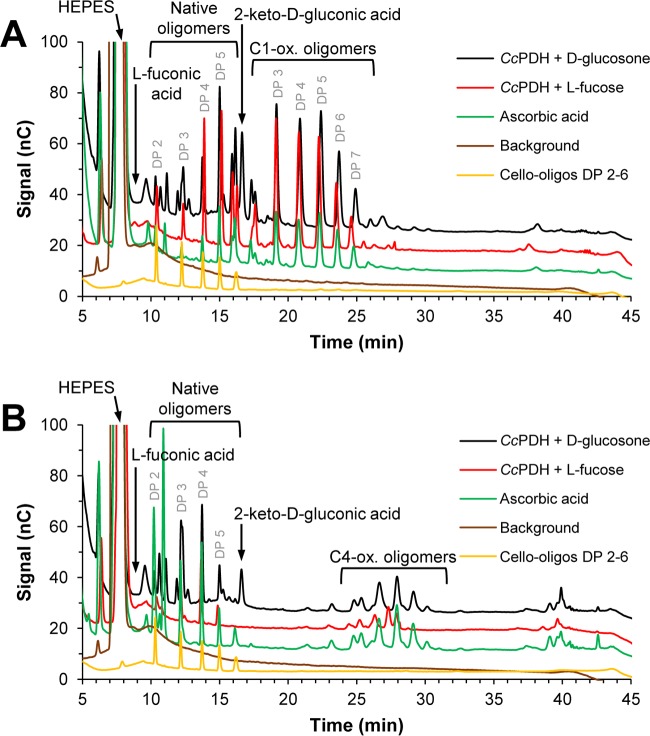
Products generated by *Nc*LPMO9F (A) and *Nc*LPMO9C (B) from PASC in the presence of various electron donating systems. Reaction mixtures containing 0.2% (wt/vol) PASC in 20 mM HEPES buffer at pH 7.0 were incubated at 30°C for 10 min. The reaction mixtures contained either 1 mM ascorbic acid (green) or 1 μM *Cc*PDH with 1 mM d-glucosone (black) or l-fucose (red) as the electron donating system. Control experiments with LPMO only, i.e., without any added component involved in electron donation, (brown) are also shown. Peak annotations are based on earlier work (see, e.g., Isaksen et al. [58]) and on control runs with standards as displayed in Fig. S1. The signal of amperometric detection is expressed in nanocoulombs (nC). Note that the response factor for l-fuconic acid is very low, explaining why there is no clearly visible peak. See Fig. S1 for more details. Numbers above peaks representing native or C-1-oxidized oligosaccharides indicate the number of sugars in the oligomer (DP, degree of polymerization); exact DP assignments for C-4-oxidized products were not possible. Cello-oligos, cello-oligosaccharides; ox., oxidizing.

**FIG 2:**
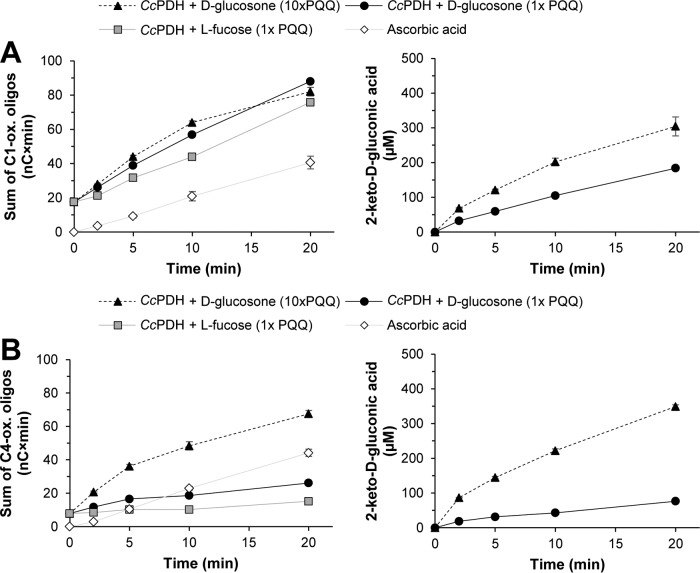
Product accumulation by *Nc*LPMO9F (A) and *Nc*LPMO9C (B) in reactions with PASC, in the presence of various electron donating systems. Reaction mixtures containing 0.2% (wt/vol) PASC in 20 mM HEPES buffer at pH 7.0 were incubated at 30°C for 20 min. The reaction mixtures contained either 1 mM ascorbic acid (white diamond) or 1 μM *Cc*PDH with 1 mM d-glucosone (black circle, solid line) or l-fucose (gray square) or 1 μM *Cc*PDH with 9 μM additional PQQ and 1 mM d-glucosone (black triangle, dashed line) as electron donating system. At *t* = 0, reactions were started by adding ascorbic acid or the *Cc*PDH substrate. In the case of the oxidized oligosaccharides (left graphs), the values provided represent the sum of the peak areas for all detectable individual oxidized species with different degrees of polymerization (3–7). *Cc*PDH products generated from d-glucosone (right graphs) were quantified using 2-keto-d-gluconic acid standards. Control reaction mixtures containing no *Cc*PDH did not show product increase over time. Control reaction mixtures containing PQQ-loaded *Cc*PDH without its substrate showed some product formation (Fig. 3), which explains why LPMO products are observed at *t* = 0. The reactions were set up in such a way that maximal background levels of oxidized cello-oligosaccharides had been reached by *t* = 0 (for details, see Fig. 3). Reactions were carried out in triplicates; error bars show the standard deviation.

**FIG 3:**
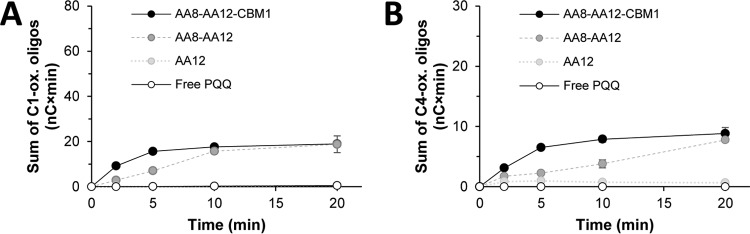
Control experiments showing the background levels of oxidized cello-oligosaccharides due to LPMO activation by free PQQ or PQQ-saturated *Cc*PDH variants in the absence of *Cc*PDH substrates. The two panels show background levels of oxidized cello-oligosaccharides generated by *Nc*LPMO9F (A) and *Nc*LPMO9C (B) due to activation by free PQQ (white circles) and PQQ-saturated *Cc*PDH variants (full-length, AA8-AA12-CBM1, black circles; two-domain, AA8-AA12, dark gray circles; single-domain, AA12, light gray circles). Reaction mixtures contained 0.2% (wt/vol) PASC, 1 μM LPMO, and 1 μM PQQ or PQQ-saturated *Cc*PDH variant in 20 mM HEPES buffer at pH 7.0 and were incubated at 30°C. Note that the reaction mixtures did not contain a substrate for *Cc*PDH. Reactions were initiated at *t* = 0 by the addition of PQQ or PQQ-saturated *Cc*PDH variant.

Figure 1 shows that when supplied with substrate, *Cc*PDH can activate *Nc*LPMO9C (NCU02916), a C-4-oxidizing LPMO with a C-terminal CBM1 domain, and *Nc*LPMO9F (NCU03328), a C-1-oxidizing single-domain LPMO (8, 24, 37). LPMO9 activity was monitored by measuring the activity against phosphoric acid-swollen cellulose (PASC), and the released oligomeric products were detected by high-performance anion-exchange chromatography (HPAEC). In the presence of ascorbic acid, *Nc*LPMO9F generated C-1-oxidized cello-oligosaccharides, while *Nc*LPMO9C generated C-4-oxidized cello-oligosaccharides. Similar product profiles were observed when supplying the reaction mixtures with *Cc*PDH and either d-glucosone or l-fucose (Fig. 1), showing that product specificity was not affected by varying the electron donor. Analysis of product formation over time (Fig. 2) revealed that *Nc*LPMO9F performed slightly better when activated by *Cc*PDH than when activated by ascorbic acid, whereas *Nc*LPMO9C performed better when activated with ascorbic acid. Control reactions with free PQQ (without *Cc*PDH) showed negligible (but larger than zero) amounts of LPMO products (Fig. 3), whereas the addition of PQQ-loaded *Cc*PDH as such (i.e., without a PDH substrate) did lead to significant product formation (Fig. 3), explaining why the product formation curves shown in Fig. 2 start from product levels higher than 0 at time (*t*) = 0 (see Fig. 2 legend).

We also tested the effect of additional PQQ on the LPMO-*Cc*PDH system (Fig. 2). For both LPMOs, the addition of free PQQ, a potential redox mediator, enhanced LPMO activity, leading to an increase in the formation of oxidized products by the enzymes. Interestingly, free PQQ also promoted *Cc*PDH activity (Fig. 2A and B, right), underpinning the strong coupling between the LPMO and the PDH reactions. The effect of adding free PQQ, in addition to PQQ-loaded *Cc*PDH, on LPMO activity was clearly larger in the case of *Nc*LPMO9C, which is the LPMO that seemed less well activated by *Cc*PDH than by *Nc*LPMO9F. It is worth noting that PDH activity is LPMO dependent (Fig. 2A and B, right) and that this dependency disappeared when additional PQQ was added. In the presence of additional PQQ, d-glucosone oxidation levels were similar in the two reactions. This suggests that the efficiency of electron transfer from *Cc*PDH to an LPMO varies between LPMOs and that limitations in this transfer may be alleviated by redox mediators.

### The functions of the AA8, AA12, and CBM1 domains in activating LPMOs.

In order to investigate whether *Cc*PDH activates an LPMO only through its AA8 domain or also through its AA12 domain, we produced three variants of *Cc*PDH, the full-length *Cc*PDH (AA8-AA12-CBM1), the AA8-AA12 two-domain enzyme, and the AA12 single-domain enzyme, and studied their ability to oxidize d-glucosone and activate the LPMOs in reactions similar to those described above.

**FIG 4:**
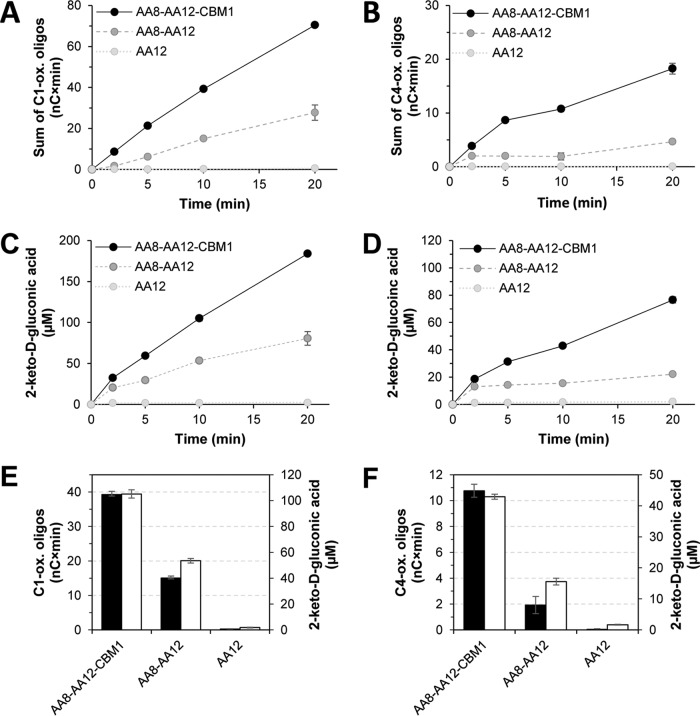
The effect of individual *Cc*PDH domains on *Cc*PDH activity and on the activation of *Nc*LPMO9F (A, C, and E, left graphs) and *Nc*LPMO9C (B, D, and F, right graphs). (A to D) Curves for the LPMO (A and B) and the PDH (C and D) reactions, respectively. Progress curves for three *Cc*PDH domain variants are shown: solid black line, full-length (AA8-AA12-CBM1); dashed dark gray line, two-domain (AA8-AA12); dotted light gray line, single-domain (AA12)variants. Reaction mixtures contained 0.2% (wt/vol) PASC, 1 μM LPMO, and 1 μM *Cc*PDH variant in 20 mM HEPES buffer at pH 7.0 and were incubated at 30°C. Reactions were initiated at *t* = 0 by the addition of d-glucosone (1,000 μM), which initiates the *Cc*PDH reaction and, thus, formation of 2-keto-d-gluconic acid. (E and F) Accumulation of oxidized cello-oligosaccharides (sum of peak areas; black bars) and 2-keto-d-gluconic acid (quantified using a standard; white bars) after 10 min. The values shown have been corrected for the product levels at *t* = 0 (see *t* = 20-min levels in Fig. 3 and legend to Fig. 2 for further explanation). Reactions were carried out in triplicate; error bars show the standard deviation.

Figure 4 shows that the activity of the AA12 domain depended strongly on the presence of the AA8 domain. Removal of the AA8 domain almost completely abolished LPMO activity and AA12 activity. It has been shown previously that the presence of an electron acceptor, such as phenazine methosulfate (PMS), is crucial for the dehydrogenase activity of an isolated AA12 domain (38). The fact that the single AA12 domain oxidized d-glucosone to a much lower extent than the full-length enzyme shows that, in the absence of the AA8 domain, the presence of an electron-accepting LPMO is not sufficient to (efficiently) reoxidize the active site of the AA12 domain. Accordingly, Fig. 4 also shows that the single AA12 domain cannot activate the two LPMOs. Thus, with an LPMO being the only available electron acceptor, the AA8 domain is needed for mediating electron transfer from the PQQ cofactor in the AA12 domain to the LPMO, which activates the LPMO and is essential for reoxidizing *Cc*PDH.

Surprisingly, deletion of the CBM1, leaving an AA8-AA12 protein that would seem fully capable of electron transfer, also reduced the performances of both the PDH and the LPMO in reactions with PASC, indicating that electron transfer is impaired also in this case (Fig. 4E and F). This effect of deleting the CBM1 from *Cc*PDH is similar for the two LPMOs and thus seems independent of the presence (*Nc*LPMO9C) or absence (*Nc*LPMO9F) of a CBM1 domain in the LPMO itself. Of note, the low-level background activation of LPMOs by *Cc*PDH in the absence of substrate is also reduced upon removal of the CBM (Fig. 3). These observations suggest that adsorption of *Cc*PDH to cellulose through the CBM1 domain, which would promote electron transfer to the LPMO to occur in the vicinity of the LPMO substrate, could enhance the efficiency of the electron transfer chain involving *Cc*PDH, LPMO, and substrate.

### Dose-response experiments.

**FIG 5:**
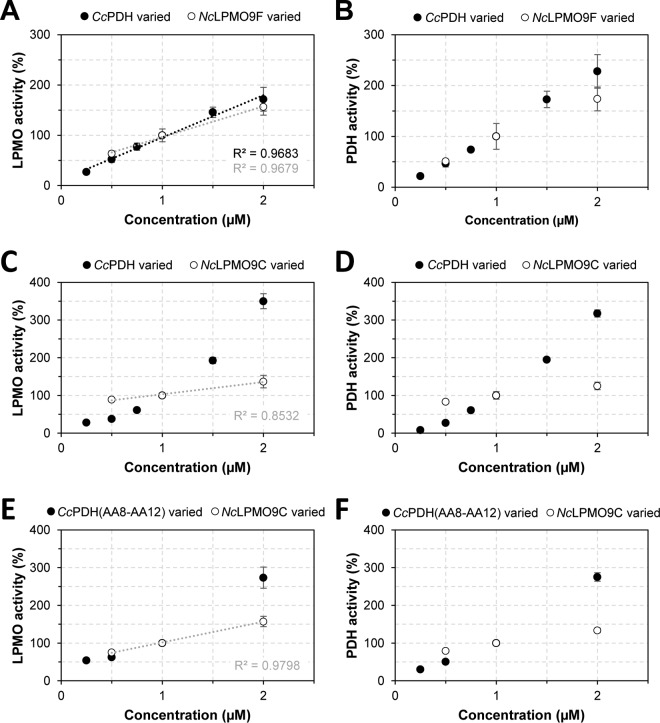
The effect of varying enzyme ratios on LPMO (A, C, and E) and PDH (B, D, and F) activity. Reaction mixtures contained 0.2% (wt/vol) PASC and 1 mM d-glucosone in 20 mM HEPES buffer (pH 7.0) and were incubated at 30°C for 10 min. The coincubated enzymes were *Nc*LPMO9F and *Cc*PDH (A and B), *Nc*LPMO9C and *Cc*PDH (C and D), and *Nc*LPMO9C and 2-domain *Cc*PDH (AA8-AA12) (E and F). In the reaction, either the PDH concentration (black symbols) or the LPMO concentration (white symbols) was varied, while the concentration of the other enzyme was kept constant at 1 μM. (A, C, and E) Relative LPMO activities, which were quantified based on the accumulation of oxidized cello-oligosaccharides. (B, D, and F) Relative PDH activities, which were quantified based on the accumulation of 2-keto-d-gluconic acid.

**FIG 6:**
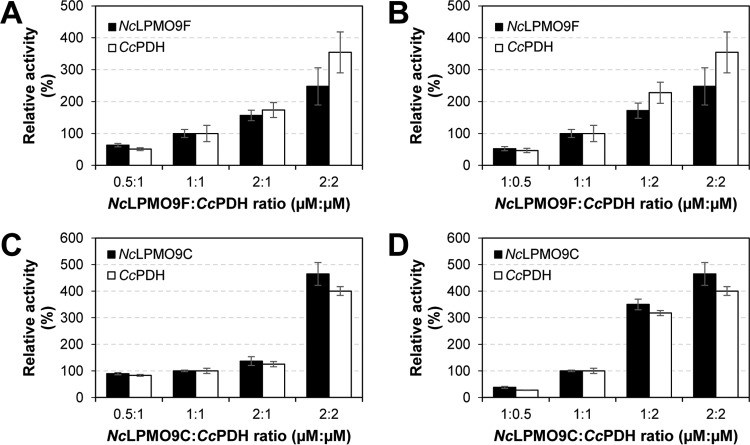
Effect of varying enzyme concentrations and ratios on LPMO and PDH activity. The graphs show a selection of the data shown in Fig. 5 and include one extra reaction (2 μM each enzyme; same reaction conditions). The coincubated enzymes were: (A and B) *Nc*LPMO9F and *Cc*PDH, primarily varying the concentration of *Nc*LPMO9F (A) or *Cc*PDH (B); and (C and D) *Nc*LPMO9C and *Cc*PDH, primarily varying the concentration of *Nc*LPMO9C (C) or *Cc*PDH (D). The black bars indicate LPMO activity, quantified by the accumulation of oxidized cello-oligosaccharide products. The white bars indicate PDH activity, quantified by the accumulation of 2-keto-d-gluconic acid.

To gain further insight into the rate-limiting steps in the *Cc*PDH-LPMO system, we coincubated *Nc*LPMO9F or *Nc*LPMO9C with *Cc*PDH while varying the *Nc*LPMO9-to-*Cc*PDH ratios and keeping the other reaction parameters constant. In the reactions with *Nc*LPMO9F and *Cc*PDH, the amount of oxidized products released by the two enzymes depended equally on the two enzymes and correlated linearly and with their concentrations (Fig. 5A and B and 6A and B). The *Nc*LPMO9C-*Cc*PDH system showed a remarkably different pattern. While variation in the *Cc*PDH concentration had a large and superstoichiometric effect on both the PDH and LPMO activities, the effects of varying the *Nc*LPMO9C concentration were much smaller (Fig. 5C and D and 6C and D). Similar trends were observed when using the CBM1-free variant of *Cc*PDH (Fig. 5E and F).

## DISCUSSION

Enzymatic electron donors for LPMO9s can act in different manners. Single-domain enzymes transfer electrons to LPMO9s through diphenol/quinone mediators and, albeit less efficiently, sometimes also directly (20, 25). Multidomain enzymes, such as *Mt*CDH from Myriococcum thermophilum, can activate LPMO9s through diphenol/quinone mediators or through an appended AA8 cytochrome domain (7, 17, 20). Measurements of electron transfer by *Mt*CDH have shown that the AA8 cytochrome domain is essential for electron transfer from *Mt*CDH to an LPMO9, whereas electron transfer directly from the AA3_1 dehydrogenase domain is absent or very slow (24). Notably, the catalytic rates of LPMOs are orders of magnitude lower than the measured rates of CDH reoxidation by an LPMO (20), meaning that a lack of measurable electron transfer does not necessarily mean a complete inability to drive LPMO activity.

*Cc*PDH has a multidomain structure similar to that of CDH, except that its central catalytic domain is a PQQ-dependent AA12 dehydrogenase instead of an FAD-dependent AA3_1 dehydrogenase domain. Figure 2 shows that when supplied with substrate, *Cc*PDH indeed can drive the LPMO reaction, and it also shows that electron consumption by the LPMO reaction is required to maintain *Cc*PDH activity. Importantly, a comparison of the results obtained with *Nc*LPMO9C and *Nc*LPMO9F showed that *Nc*LPMO9F is more easily activated than *Nc*LPMO9C. However, when supplying a potential redox mediator (free PQQ), the two *Cc*PDH-LPMO systems work approximately equally quickly, as judged from the observed *Cc*PDH activity (Fig. 2, right; note that the amount of added PQQ is much smaller than the amount of generated product, meaning that the PQQ acts as a mediator, not as a reductant). These observations indicate that in the absence of a mediator, direct reoxidation of *Cc*PDH by *Nc*LPMO9C is slower than reoxidation by *Nc*LPMO9F. In this respect, it is worth noting that analyses of the rates of reoxidation of two CDHs from Neurospora crassa by LPMOs showed that, depending on the conditions and the CDH, those rates are up to one order of magnitude lower for *Nc*LPMO9C than for *Nc*LPMO9F (20) (*Nc*LPMO9F gave the fastest kinetics of the four tested *Nc*LPMOs). Thus, LPMOs seem to differ considerably when it comes to their ability to interact with the AA8 cytochrome domain attached to AA3 and AA12 dehydrogenases.

Figure 4 shows that the AA8 cytochrome domain is crucial in driving the LPMO reaction. It has previously been suggested that the heme *b* propionate of the AA8 domain in *Mt*CDH (31.8% sequence identity with the AA8 domain of *Cc*PDH) may have favorable interactions with the copper active site of *Nc*LPMO9F (24), and nuclear magnetic resonance (NMR) experiments with *Nc*LPMO9C and *Mt*CDH have shown that there indeed are specific interactions between the cytochrome domain and the catalytic center of the LPMO (39). To date, the AA8 domain has been found in three types of fungal proteins, namely, AA3_1 CDH, AA12 PDH, and cellulose-binding cytochrome *b*_562_ (CBCyt *b*_562_; an N-terminal AA8 domain fused to a C-terminal CBM1), and these AA8 domains are highly similar to each other in terms of amino acid sequence and spectral, biochemical, and electrochemical features (34, 40, 41). PDHs, CDHs, and CBCyt *b*_562_ are extracellular proteins, can bind to cellulose, and often carry a cellulose-binding CBM1 module. This may be taken to suggest that these AA8 domains have evolved to enable interprotein electron transfer on cellulose surfaces in order to facilitate the extracellular redox pathways of fungal plant cell wall degradation.

Cellulose binding through a CBM1 enhances the efficiency of many plant cell wall-degrading enzymes, such as cellulases and hemicellulases, LPMOs, and CDHs (34, 42–45). On the other hand, the catalytic modules of these enzymes also have affinity for the substrate. Basidiomycetous CDHs, generally lacking a CBM1, possess a putative substrate-binding site located in the flavin-dependent AA3_1 domain (27, 46–48). The AA3_1 domains of ascomycetous CDHs lack this binding site (49), and these CDHs usually contain a C-terminal CBM1. A possible role of this CBM in promoting LPMO activation by CDH has not yet been assessed. Similarly to ascomycetous CDHs, *Cc*PDH possesses a CBM1 at the C terminus, which enables its binding to both amorphous and crystalline celluloses (34). Figure 4 shows that the CBM1 domain of *Cc*PDH is of considerable importance for the efficiency of the *Cc*PDH-LPMO system, since the presence of CBM1 in *Cc*PDH had a positive effect on both LPMO and *Cc*PDH activities. This surprising and novel finding indicates that it is beneficial that electron transfer leading to the activation of the LPMO takes place in the vicinity of the crystalline cellulose surface. It is conceivable that LPMO action is enhanced by increasing the proximity between the substrate and the electron source for LPMOs. Likewise, *Cc*PDH activity may be enhanced by the presence of an electron acceptor that will rapidly oxidize cellulose and be available for accepting new electrons. For the LPMO, activation close to the substrate also may have additional beneficial effects, since it has been shown that activated (reduced) LPMOs that are not bound to substrate suffer from oxidative self-inactivation (50). It is noteworthy that the effect of removing the CBM1 from *Cc*PDH was largest for *Nc*LPMO9C, which contains a CBM1, when acting on its preferred substrate, PASC. It is possible that LPMOs carrying a CBM1 benefit more from the presence of a CBM1 in *Cc*PDH than single-domain LPMOs, as the CBM1 domain directs the PDH on parts of the cellulose surface where *Nc*LPMO9C is associated, thus further increasing the interaction efficiency of the two enzymes.

Recently, it has been proposed that LPMOs may use H_2_O_2_ as a cosubstrate and even prefer H_2_O_2_ over O_2_ (51). In this scenario, stoichiometric and continuous transfer of electrons to the copper site in the LPMO is not necessary if H_2_O_2_ is supplied. Instead, the LPMO is primed by a one-electron reduction of the copper, after which the primed enzyme can carry out multiple reactions using H_2_O_2_. In the absence of externally supplied H_2_O_2_ but in the presence of reductant, H_2_O_2_ can be generated by the LPMO itself (52) or by reactions involving O_2_ and the reductant, or, as some would argue, the LPMOs can use another O_2_-based mechanism (21, 50). Although not very visible in Fig. 4, the AA12 domain can drive LPMO reactions at very low rates, analogous to what has been observed for certain flavin-dependent AA3_2 dehydrogenase domains (25). Slow reoxidation of the dehydrogenase by O_2_ and concomitant production of hydrogen peroxide, combined with LPMO reduction by low concentrations of free PQQ, or perhaps even directly by the AA12 domain itself, could explain why the AA12 domain alone can, although very slowly, drive LPMO reactions.

Our current view on LPMO catalysis is that in the absence of externally supplied H_2_O_2_ (as is the case for all reactions shown in this study) and in the presence of a reductant, the LPMO generates its own H_2_O_2_ from O_2_, which, notably, would require two electrons. Thus, no matter which LPMO mechanism one assumes, under the conditions used in this study, each LPMO catalysis consumes two electrons. This explains why the observed activities of *Cc*PDH, which needs to get rid of two electrons in each cycle, and the LPMO, which needs to acquire two electrons in each cycle, are tightly coupled. A key remaining question for all scenarios without externally added H_2_O_2_ is how and when the second electron is transferred to the catalytic center, perhaps especially for scenarios where the LPMO is bound to substrate and has a less accessible copper site. It is worth noting that transfer of the second electron likely is rate limiting, since one-electron reduction of the copper in a free LPMO (i.e., transfer of the first electron) is orders of magnitude faster than LPMO catalysis driven by, e.g., ascorbic acid (20).

The experiments with varied enzyme ratios show large differences between the *Cc*PDH-*Nc*LPMO9F and the *Cc*PDH-*Nc*LPMO9C systems. In the *Cc*PDH-*Nc*LPMO9F system, the electron transfer and enzyme catalytic rates are apparently so high that, in the concentration range used in Fig. 5, the two enzymes limit each other. In other words, as shown in Fig. 5, increased amounts of reducing equivalents generated by increasing *Cc*PDH concentrations can be absorbed by a stable amount of LPMO, while an increased demand for reducing equivalents by increasing amounts of LPMO can be supplied by a stable amount of *Cc*PDH. For the *Cc*PDH-*Nc*LPMO9C system, the situation is very different. Increasing the concentration of *Nc*LPMO9C only marginally increased LPMO and PDH activities, whereas increasing the concentration of *Cc*PDH led to a superstoichiometric increase in LPMO and PDH activities. The observation regarding the concentration of *Cc*PDH is not straightforward to explain but is likely due to a weak interaction between *Nc*LPMO9C and *Cc*PDH, as discussed above. In this case, electron transfer may be so slow that the first, presumably fast, reduction of the copper (the “priming” reduction) becomes rate limiting. Increasing the PDH concentration would then have a double effect, since more *Nc*LPMO9C molecules will become catalytically competent whereas, at the same time, production of the cosubstrate H_2_O_2_ will increase.

Small molecular reductants, such as ascorbic acid, are regularly used as an electron donor for LPMOs, while such compounds are generally unstable, may lead to side reactions, and could lead to the inactivation of LPMOs at higher concentrations (26, 51). Studies on the activity of a chitin-active LPMO have shown that stable reaction kinetics are more easily obtained using CDH (with lactose as the substrate) to deliver electrons. Unfortunately, CDH interferes with the accurate determination of cello-oligosaccharides produced by LPMOs due to oxidation of the reducing ends of C-4-oxidized and native cello-oligosaccharides. *Cc*PDH, on the other hand, is inactive against the most common sugars in biomass (such as d-glucose, as shown in Fig. S4, as well as d-xylose and d-mannose [33]) and will not oxidize the most common oligosaccharides produced by LPMO activity. Moreover, the reaction products of *Cc*PDH (i.e., 2-keto-d-gluconic acid and, to a much lesser degree, l-fuconic acid) can be distinguished from oxidized cello-oligosaccharides in HPAEC-pulsed amperometric detection (HPAEC-PAD) analyses, which allow not only proper detection of oxidized cello-oligosaccharides but also simultaneous monitoring of dehydrogenase and LPMO activities. Thus, we believe that *Cc*PDH will turn out to be a useful tool in future studies of LPMOs and associated redox enzyme systems involved in biomass degradation.

## MATERIALS AND METHODS

### Materials.

Pyrroloquinoline quinone (PQQ) disodium salt and l-fucose were purchased from Wako Pure Chemical Industries (Osaka, Japan). d-Glucosone (2-keto–d-glucose) was purchased from Santa Cruz Biotechnology (Santa Cruz, CA, USA). 2-Keto-d-gluconic acid hemicalcium salt hydrate, l-fucono-1,4-lactone, cytochrome *c* from bovine heart, and Avicel PH 101 were purchased from Sigma-Aldrich (St. Louis, MO, USA). Phosphoric acid-swollen cellulose (PASC) was prepared from Avicel as described by Wood (53).

### Preparation of enzymes.

Full-length *Cc*PDH (AA8-AA12-CBM1), truncated two-domain *Cc*PDH (AA8-AA12), and the single-domain PQQ-dependent dehydrogenase domain (AA12) were heterologously expressed in the methylotrophic yeast Pichia pastoris and purified as described previously (33). AA8-AA12 and AA12 were subjected to additional purification using a Superdex 75 gel filtration column (GE Healthcare, Sweden) run in 20 mM HEPES (pH 7.0) and 150 mM NaCl. Recombinant *Nc*LPMO9C and *Nc*LPMO9F from Neurospora crassa were prepared according to Kittl et al. (52). Protein purity was confirmed by SDS-PAGE analysis. *Nc*LPMO9C and *Nc*LPMO9F were saturated with Cu(II) by incubating enzymes with an excess of CuSO_4_ (at a 3:1 molar ratio of copper/enzyme) for 30 min at room temperature, as described previously (55). After saturation, excess CuSO_4_ was removed using PD-10 desalting columns (GE Healthcare).

Holo-forms (i.e., PQQ- and Ca^2+^-saturated forms) of *Cc*PDH and its truncated versions were prepared by incubating 20 μM enzyme with 200 μM PQQ and 2 mM CaCl_2_ for 30 min on ice, followed by removal of excess PQQ and CaCl_2_ using PD-10 desalting columns. PQQ saturation was confirmed by analyzing the enzyme activity of holo-*Cc*PDH with and without the addition of extra (5 μM) PQQ and (50 μM) CaCl_2_. The enzyme activity of *Cc*PDH was measured spectrophotometrically by monitoring the reduction of cytochrome *c* at 550 nm and 30°C (34). The reaction mixture contained 200 nM holo-enzyme, 50 mM piperazine-*N*,*N*′-bis(2-ethanesulfonic acid) (PIPES)-NaOH (pH 7.0), 1 mM l-fucose, and 50 μM cytochrome *c* in a total volume of 500 μl. The addition of extra PQQ and CaCl_2_ to the putatively saturated enzyme had no effect on enzyme activity, indicating that the holo-enzyme was fully saturated (Fig. S5).

Protein concentrations were measured using the Bradford method (Bio-Rad's protein assay kit; Bio-Rad Laboratories, Hercules, CA, USA) with bovine serum albumin as a standard.

### Activity assays.

For detecting LPMO activity, 1 μM LPMO was incubated with 0.2% (wt/vol) PASC in 20 mM HEPES buffer (pH 7.0) at 30°C for 10 min. All reaction mixtures contained 100 μM CaCl_2_. As an electron donor, either 1 mM ascorbic acid or 1 μM *Cc*PDH holoenzyme supplemented with 1 mM d-glucosone or l-fucose was supplied, unless otherwise stated. Control reactions were set up in the same manner, leaving out one or more components, as indicated in the figure legends. In the dose-response experiments, the concentrations of *Nc*LPMO9s and *Cc*PDHs were varied between 0.25 and 2.0 μM. Reactions were stopped by boiling for 5 min. All reaction products were analyzed by high-performance anion-exchange chromatography (HPAEC) using a Dionex ICS 3000 system (Dionex, Sunnyvale, CA, USA) equipped with a CarboPac PA1 precolumn (2 by 50 mm), a CarboPac PA1 main column (2 by 250 mm), and a detector for pulsed amperometric detection (PAD), applying a 50-min gradient that has been described previously (56). Chromatograms were collected and analyzed with Chromeleon 7.0. In the dose-response experiments, the relative activity of *Cc*PDH was calculated as the ratio of peak areas for 2-keto-d-gluconic acid in the given reaction sample and in the analogous sample from the reaction mixture containing 1 μM LPMO and 1 μM *Cc*PDH. In the case of the LPMOs, the corresponding ratios were calculated for each oxidized oligosaccharide (with different degree of polymerization), and the mean of those ratios is presented as relative activity. In the case of *Nc*LPMO9C, which produces large amounts of (partly on column-generated) native oligosaccharides (57), relative activities were calculated using peaks for C-4-oxidized products. The corresponding values were also calculated using peaks for the native products, which generally corresponded well with the values based on oxidized products, adding confidence to the results. All experiments were performed in triplicate.

## Supplementary Material

Supplemental material
