# Supplementary material for: The Pyrroloquinoline-Quinone-Dependent Pyranose Dehydrogenase from Coprinopsis cinerea Drives Lytic Polysaccharide Monooxygenase Action
Source: Appl Environ Microbiol. 2018 May 17;84(11):e00156-18. doi: 10.1128/AEM.00156-18 (PMC5960967; doi:10.1128/AEM.00156-18)
Supplement: Supplemental material [file supp_84_11_e00156-18__index.html]

Supplemental material 

# The Pyrroloquinoline-Quinone-Dependent Pyranose Dehydrogenase from Coprinopsis cinerea Drives Lytic Polysaccharide Monooxygenase Action

## Supplemental material

- Supplemental file 1 -

  Identification of products generated by *Cc*PDH from d-glucosone and l-fucose with HPAEC-PAD (Fig. S1); products generated by *Nc*LPMO9F or *Nc*LPMO9C from PASC using *Cc*PDH as electron donor (Fig. S2); HPAEC-PAD chromatograms showing lack of *Nc*LPMO9F or *Nc*LPMO9C activity by boiled *Cc*PDH products (Fig. S3); HPAEC-PAD chromatograms showing the lack of activity of *Cc*PDH on d-glucose (Fig. S4); the effect of adding extra PQQ and Ca2+ on the activity of holo-*Cc*PDH (Fig. S5).

  PDF, 454K
