# Supplementary material for: The Pyrroloquinoline-Quinone-Dependent Pyranose Dehydrogenase from Coprinopsis cinerea Drives Lytic Polysaccharide Monooxygenase Action
Source: Appl Environ Microbiol. 2018 May 17;84(11):e00156-18. doi: 10.1128/AEM.00156-18 (PMC5960967; doi:10.1128/AEM.00156-18)
Supplement: Supplemental material [file AEM.00156-18_zam011188534s1.pdf]

# The pyrroloquinoline-quinone dependent pyranose dehydrogenase from *Coprinopsis cinerea* (*Cc*PDH) drives lytic polysaccharide monooxygenase (LPMO) action

Anikó Várnai[a,*], Kiwamu Umezawa[b,‡,*], Makoto Yoshida[b,†,#], Vincent G. H. Eijsink[a,†,#]

[a]Faculty of Chemistry, Biotechnology and Food Science, Norwegian University of Life Sciences (NMBU), 1432 Ås, Norway

[b]Department of Environmental and Natural Resource Science, Tokyo University of Agriculture and Technology, Tokyo 183-8509, Japan

## SUPPLEMENTAL INFORMATION

## CONTENTS

- **Figure S1.** Identification of products generated by *Cc*PDH from D-glucosone and L-fucose with HPAEC-PAD.
- **Figure S2.** Products generated by *Nc*LPMO9F or *Nc*LPMO9C from PASC using *Cc*PDH as electron donor.
- **Figure S3.** HPAEC-PAD chromatograms showing lack of *Nc*LPMO9F or *Nc*LPMO9C activity by boiled *Cc*PDH products.
- **Figure S4.** HPAEC-PAD chromatograms showing the lack of activity of CcPDH on D-glucose.
- **Figure S5.** The effect of adding extra PQQ and $Ca^{2+}$ on the activity of holo-CcPDH.

**FIGURE S1**

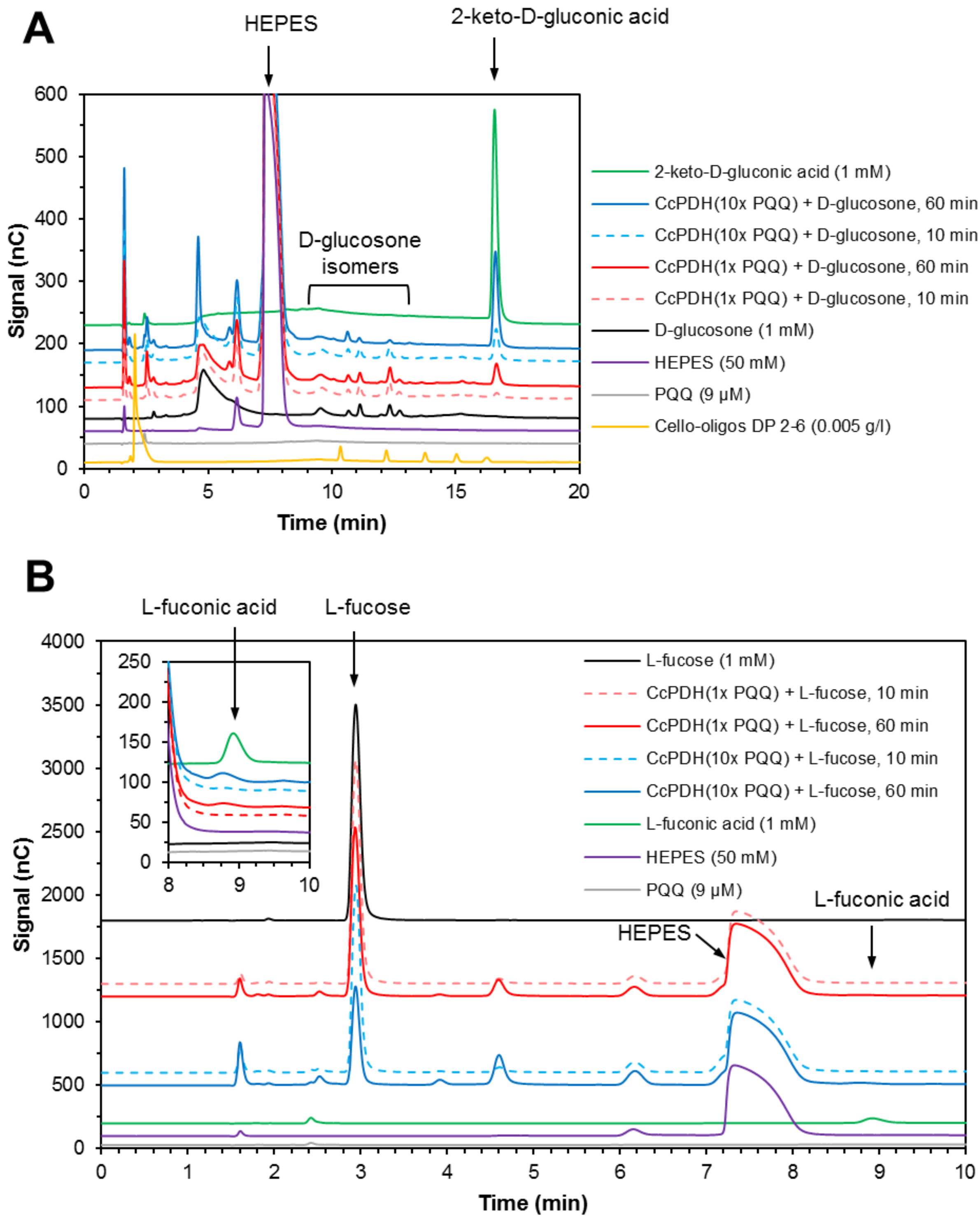


**Figure S1.** Identification of products generated by *Cc*PDH from (A) D-glucosone and (B) L-fucose with HPAEC-PAD. For reaction conditions, see Materials and methods. The retention times of the characteristic reaction products are 16.6 min (2-keto-D-gluconic acid generated from D-glucosone; Panel A) and 8.9 min (L-fuconic acid, generated from L-fucose; panel B). "1x PQQ" means PQQ-saturated *Cc*PDH (1 µM), whereas "10x PQQ" means that an additional 9 µM of PQQ was added to the reaction mixture. The insert in panel B shows the L-fuconic acid peak; note that the response factor for this compound is so low that it is hardly detectable in most experiments.

**FIGURE S2**

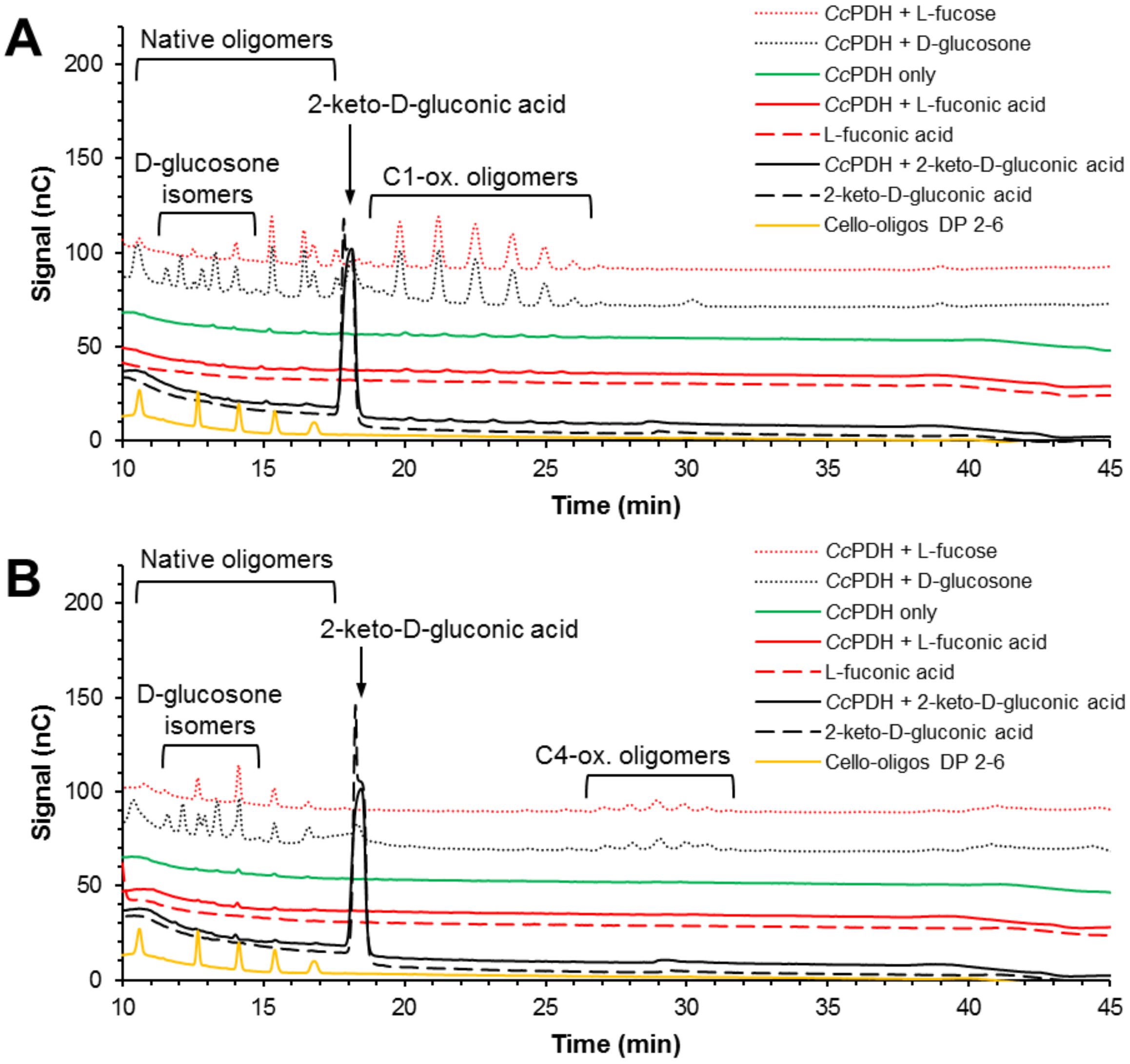


**Figure S2.** Products generated by (A) *Nc*LPMO9F or (B) *Nc*LPMO9C from PASC using *Cc*PDH as electron donor. The figure shows that the LPMOs released oxidized oligosaccharides only when *Cc*PDH and its substrate, D-glucosone or L-fucose, were supplied together. Neither 2-keto-D-gluconic acid or L-fuconic acid, the corresponding products of *Cc*PDH, were able to activate the LPMOs, independently of the presence of *Cc*PDH. Reaction mixtures contained 0.2% (w/v) PASC in 20 mM HEPES buffer at pH 7.0 and were incubated at 30 °C for 10 min. The reactions contained 1 µM *Cc*PDH with 1 mM D-glucosone (black dotted line) or L-fucose (red dotted line) as electron donating system. Control experiments included 1 µM *Cc*PDH with 1 mM 2-keto-D-gluconic acid (black solid line) or L-fuconic acid (red solid line), 1 µM *Cc*PDH only (green line), 1 mM 2-keto-D-gluconic acid only (black dashed line) and 1 mM L-fuconic acid only (red dashed line).

**FIGURE S3**

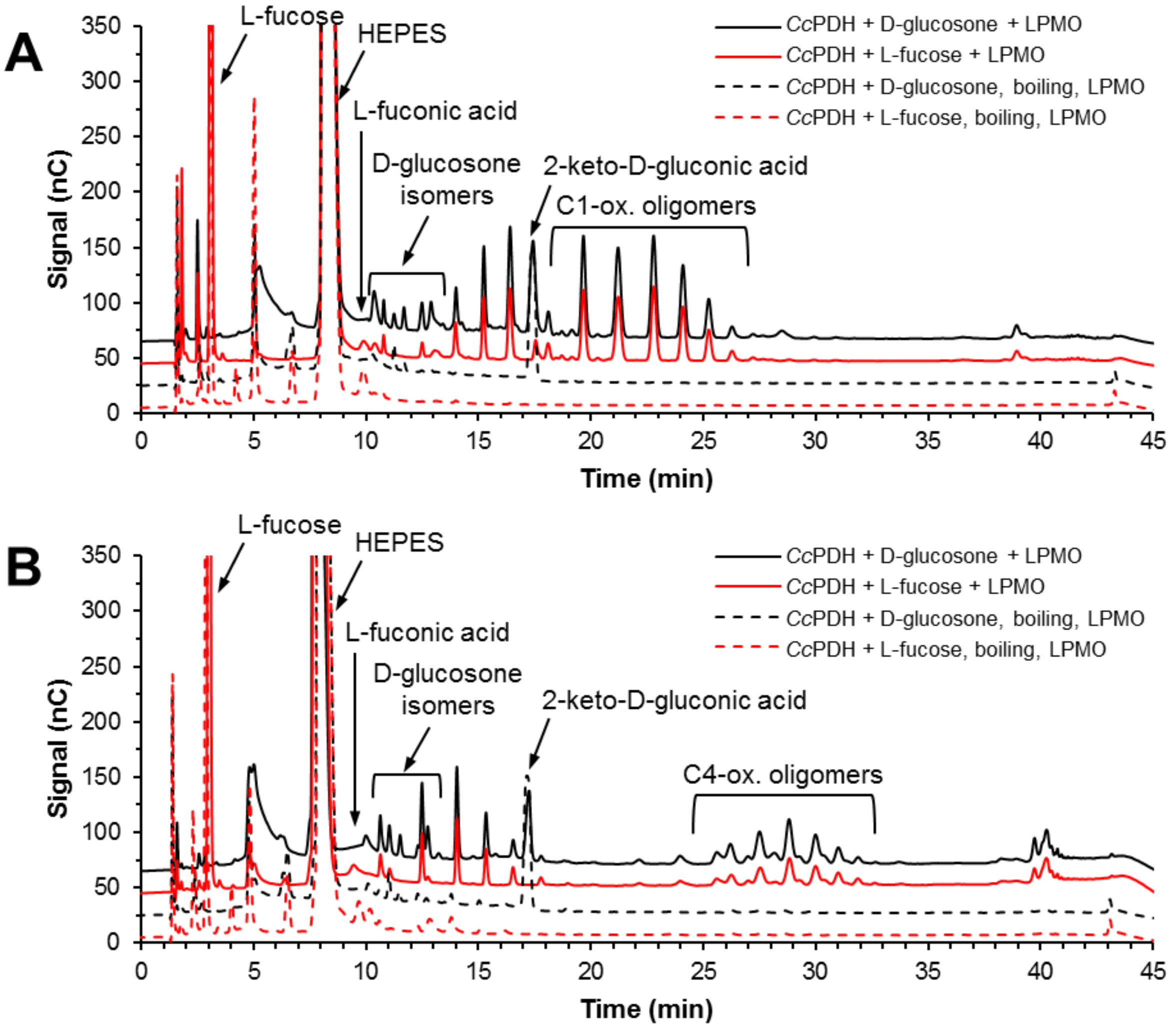


**Figure S3.** HPAEC-PAD chromatograms showing lack of (A) *Nc*LPMO9F or (B) *Nc*LPMO9C activity by boiled *Cc*PDH products. Products generated by (A) *Nc*LPMO9F or (B) *Nc*LPMO9C from PASC, using *Cc*PDH as electron donor or boiled *Cc*PDH products. The LPMOs were co-incubated with *Cc*PDH and D-glucosone (black solid line) or *Cc*PDH and L-fucose (red solid line), or with a boiled reaction mixture of *Cc*PDH pre-incubated with D-glucosone (black dashed line) or L-fucose (red dashed line). Reaction mixtures (yielding the solid lines) contained 0.2% (w/v) PASC, 1 µM LPMO and 1 µM *Cc*PDH in 20 mM HEPES buffer at pH 7.0 and were supplemented with 10 mM $CaCl_2$, 1 mM PQQ and 1 mM D-glucosone (black) or L-fucose (red) and were incubated for 10 min at 30 °C. Reactions done to assess the effect of possibly unknown additional reaction products of *Cc*PDH were incubated at 30 °C for 10 min and boiled for 5 min prior to addition of 1 µM LPMO, followed by another incubation at 30 °C for 10 min.

**FIGURE S4**

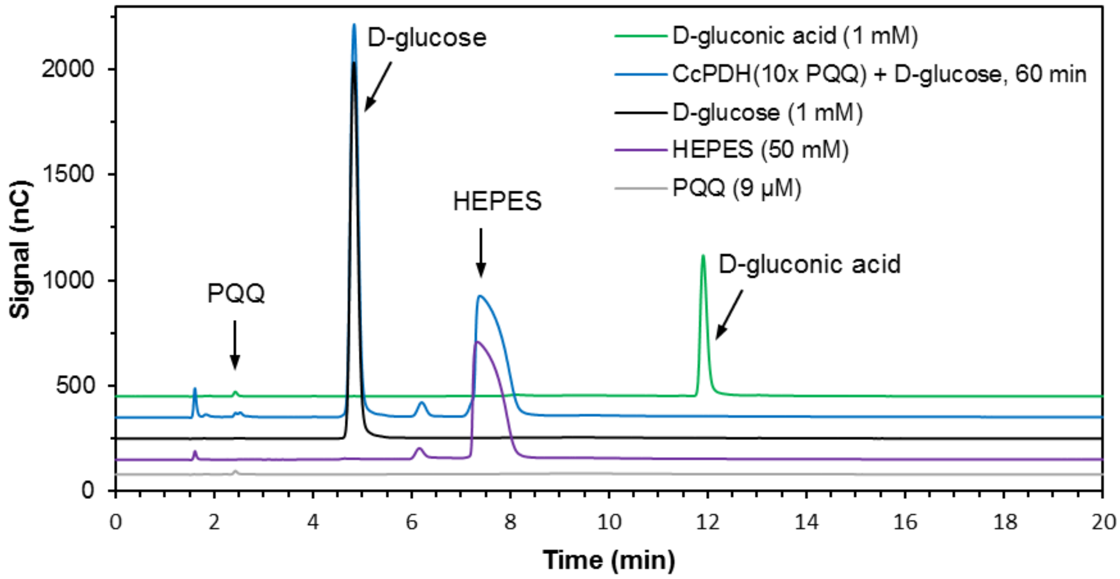


**Figure S4.** HPAEC-PAD chromatograms showing the lack of activity of *Cc*PDH on D-glucose. Comparison of the four standards with the reaction mixture obtained after incubating *Cc*PDH with 1 mM D-glucose and an additional 9 µM of PQQ in 50 mM HEPES pH 7.0, at 30 °C, for 60 minutes, shows that glucose had not been converted by *Cc*PDH.

**FIGURE S5**

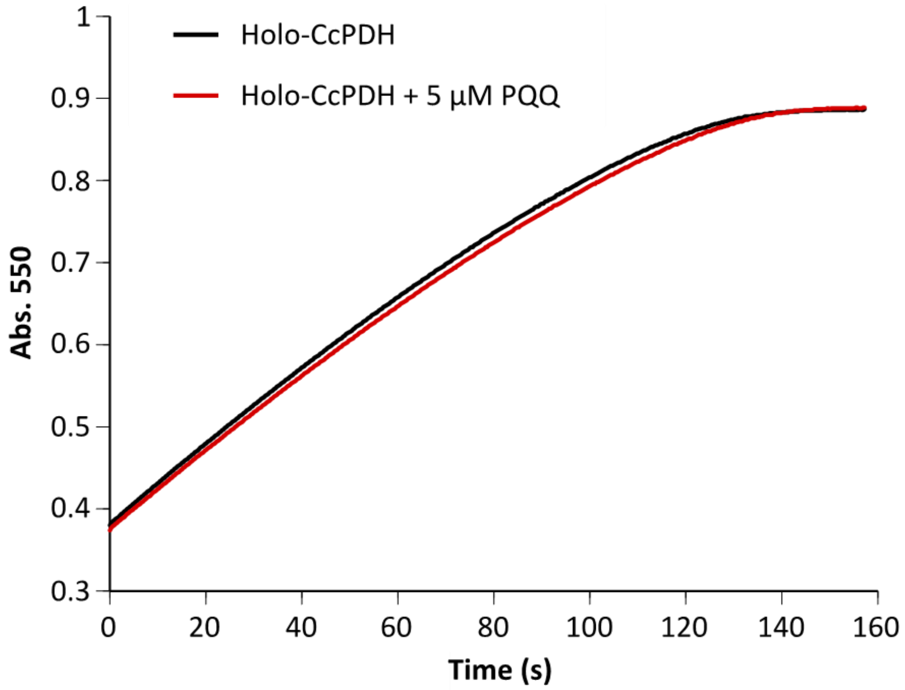


**Figure S5.** The effect of adding extra PQQ and $Ca^{2+}$ on the activity of holo-*Cc*PDH. The curves show reduction of cytochrome *c* over time in reactions containing 200 nM presumably saturated *Cc*PDH and 50 µM cytochrome *c* in 50 mM PIPES-NaOH (pH 7.0), 1 mM L-fucose. The curve labeled “+ 5 µM PQQ” shows results for a similar reaction mixture which was supplemented with additional PQQ and $CaCl_2$ (50 µM). The fact that the two curves are identical indicates that the presumably saturated *Cc*PDH indeed was saturated.
